# Adrenomedullin: its double-edged sword during sepsis slices yet again

**DOI:** 10.1186/2197-425X-2-1

**Published:** 2014-01-02

**Authors:** Matthijs Kox, Peter Pickkers

**Affiliations:** Intensive Care Medicine, Radboud University Medical Centre, Nijmegen, 6500, HB the Netherlands; Nijmegen Institute for Infection, Inflammation and Immunity (N4i), Nijmegen, 6500, HB The Netherlands

In this first issue of *Intensive Care Medicine Experimental*, two related studies describe the effects of antibodies directed against adrenomedullin (ADM), a vasoactive peptide that is released from various tissues during systemic inflammation, in murine sepsis.

ADM, derived from a larger precursor peptide (pro-ADM), is released into the circulation during systemic inflammation, and the highest plasma concentrations are measured in patients with septic shock[1]. With a plasma half-life of approximately 20 min, it exerts multiple effects of which the most relevant appears to be vasodilation[2]. The consequent clinical sequelae of increased ADM concentrations are far from clear. While elevated ADM levels are associated with impaired outcome in sepsis patients[1] and the measurement of the circulating pro-peptide of ADM might serve as a marker to assess disease severity and predict mortality[3], administration of exogenous ADM actually improved outcome in animal models of septic shock[4].

Intriguingly, in this issue of *Intensive Care Medicine Experimental*, two studies demonstrate beneficial effects of anti-ADM antibodies administered in a murine cecal ligation and puncture (CLP) model[5, 6]. These studies represent the first attempts at antibody targeting of the ADM cascade *in vivo*. Struck et al.[5] describe the development of monoclonal antibodies directed against various epitopes of ADM and their ability to attenuate sepsis-induced mortality in severe murine sepsis (100% control group mortality within 2 to 3 days). The severity of this model means that any long-term deleterious or beneficial effects of the antibody remain unknown. Notably, all antibodies directed against ADM improved survival. Of interest, the antibody against the N-terminal moiety of ADM prevented mortality most effectively, while it inhibited ADM agonistic activity by only 25%. Wagner et al.[6] performed a more mechanistic study into the effects of HAM1101, the antibody targeted against the N-terminal part of ADM, in a resuscitated murine CLP model. They show that HAM1101 improves vasoactive responsiveness to catecholamines, attenuates systemic inflammation, and improves kidney function. The beneficial effects on the kidneys are likely attributable to reduced iNOS expression and nitrotyrosine formation in HAM1101-treated animals. The effects on inflammatory pmeters are surprising as ADM itself has also been reported to inhibit inflammation[7]. In summary, previous work has established that high endogenous levels of ADM correlate with poor outcomes and that exogenous administration improved outcomes, while now it is reported that antagonism of ADM using antibodies improves outcome in an animal sepsis model.

How can we explain these apparently conflicting results? We naturally tend to prefer simple linear reasoning. However, reality, especially in the case of (patho)physiology, is more complex. ADM-mediated effects appear to represent yet another example of this fact. Timing and the magnitude of increase in ADM may be especially relevant in understanding its clinical effects in sepsis. In this respect, it is important to recognize that Struck et al. found that the least efficacious antibody in terms of antagonistic activity exerted the largest beneficial effect on survival. One could argue that other non-ADM-dependent effects of the antibody may play a role. However, it could also indicate that only very high concentrations of ADM exert deleterious effects, while modest increases of this peptide are beneficial. In addition to concentration-dependent and time-dependent effects, the specific clinical condition of sepsis, e.g., hyper- vs hypodynamic sepsis, is also likely to play a role in the observed discrepancies. It is clear that the path to clinical relevance at the bedside is long and bumpy, and based on the above described results, it is already clear that in sepsis patients, it will be a difficult task to define the therapeutic window of opportunity to modulate the ADM pathway. Nevertheless, further investigation of the effects of modulating this pathway is clearly warranted.
